# The model of litter size reduction induces long‐term disruption of the gut‐brain axis: An explanation for the hyperphagia of Wistar rats of both sexes

**DOI:** 10.14814/phy2.15191

**Published:** 2022-02-10

**Authors:** Vanessa S. T. Rodrigues, Egberto G. Moura, Thamara C. Peixoto, Patricia N. Soares, Bruna P. Lopes, Iala M. Bertasso, Beatriz S. Silva, S. S. Cabral, G. E. G. Kluck, G. C. Atella, P. L. Trindade, J. B. Daleprane, Elaine Oliveira, Patricia Cristina Lisboa

**Affiliations:** ^1^ Laboratory of Endocrine Physiology Biology Institute State University of Rio de Janeiro Rio de Janeiro Brazil; ^2^ Laboratory of Lipids and Lipoprotein Biochemistry Biochemistry Institute Federal University of Rio de Janeiro Rio de Janeiro Brazil; ^3^ Laboratory for studies of Interactions between Nutrition and Genetics Nutrition Institute Rio de Janeiro State University Rio de Janeiro Brazil

**Keywords:** microbiota, obesity, overnutrition, SCFAs, small litter, vagus nerve

## Abstract

The gut microbiota affects the host's metabolic phenotype, impacting health and disease. The gut‐brain axis unites the intestine with the centers of hunger and satiety, affecting the eating behavior. Deregulation of this axis can lead to obesity onset. Litter size reduction is a well‐studied model for infant obesity because it causes overnutrition and programs for obesity. We hypothesize that animals raised in small litters (SL) have altered circuitry between the intestine and brain, causing hyperphagia. We investigated vagus nerve activity, the expression of c‐Fos, brain‐derived neurotrophic factor (BDNF), gastrointestinal (GI) hormone receptors, and content of bacterial phyla and short‐chain fatty acids (SCFAs) in the feces of adult male and female Wistar rats overfed during lactation. On the 3rd day after birth, litter size was reduced to 3 pups/litter (SL males or SL females) until weaning. Controls had normal litter size (10 pups/litter: 5 males and 5 females). The rats were killed at 5 months of age. The male and female offspring were analyzed separately. The SL group of both sexes showed higher food consumption and body adiposity than the respective controls. SL animals presented dysbiosis (increased *Firmicutes*, decreased *Bacteroidetes*) and had increased vagus nerve activity. Only the SL males had decreased hypothalamic GLP‐1 receptor expression, while only the SL females had lower acetate and propionate in the feces and higher CCK receptor expression in the hypothalamus. Thus, overfeeding during lactation differentially changes the gut‐brain axis, contributing to hyperphagia of the offspring of both sexes.

## Graphical abstract

Litter size reduction causes vagal hyperactivity in both sexes at adulthood. Both sexes presented gut dysbiosis. Early overfeeding is able to program parameters of the gut‐brain axis and impact the eating pattern in adult life.

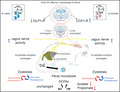

## 1 INTRODUCTION

Food intake is finely regulated by a complex system that integrates the central nervous system (CNS) with the periphery. In the CNS, there are specific regions specializing in the regulation of appetites, such as the arcuate nucleus (ARC) of the hypothalamus, which is controlled by leptin, insulin, and gastrointestinal (GI) peptide receptors (Schwartz et al., 2000). Additionally, in the brain stem, the nucleus tractus solitarii (NTS), which, in addition to having the same hormone receptors found in the hypothalamus, also receives nerve inputs from the vagal afferent fibers from the viscera (Travagli et al., 2006). After reaching the NTS, peripheral information is processed, and signals from second‐order neurons are sent to the ARC to control hunger and satiety (Van Bloemendaal et al., 2014). Additionally, hedonic control by the nuclei of the mesocorticolimbic dopaminergic system (Baik, 2013) and the nuclei of the endocannabinoid system (Valassi et al., 2008) is important for food intake.

Several signals from the gastrointestinal tract (GIT) establish a gut‐brain connection, in which we are interested in 2 pathways: (1) neurotransmitters, such as glutamate, via the vagus nerve and (2) orexigenic or anorexigenic GI hormones, such as ghrelin, cholecystokinin (CCK), glucagon‐like peptide 1 (GLP‐1), and peptide YY (PYY; Valassi et al., 2008). This interaction represents only a part of a complex network that constitutes the gut‐brain connection responsible for modulating food intake. Also, gut‐brain connection includes direct nutrient signaling, bacterial/inflammatory signaling, and inputs via the spinosolitary tract.

The GIT has its own nervous system, the enteric nervous system (ENS), which has a certain independence from the CNS (Mukhtar et al., 2019). The development of the ENS depends on BDNF (Liu, 2018); its central and peripheral expression is affected by perinatal nutritional changes (Coupé et al., 2009; Fox & Biddinger, 2012). In addition, the GIT acts as a neuroendocrine organ that produces several neurotransmitters and peptides that control food intake (Santonicola et al., 2019). The nerve connection between the CNS and GIT is bidirectional and is performed through the vagus nerve. This nerve is composed of three different types of fibers: A fiber, myelinated afferent, B fiber, myelinated efferent, and C fiber, unmyelinated afferent. Anatomically, the vagus nerve is composed predominantly of afferent fibers; it has 60%–80% afferent fibers (sensory) that carry visceral information to the center (Yuan & Silberstein, 2016), for instance, via glutamate for NTS (Minaya et al., 2018; Sykes et al., 1997; Travagli et al., 2006). The remaining fibers, about 20%, constitute the efferent fibers (motor) that control the cardiovascular, respiratory, immune, and endocrine systems (Browning et al., 2017); in the intestine, the efferent response of this nerve is mediated by acetylcholine (Boeckxstaens, 2013). The information sent to the CNS is transmitted by mechanical stimuli, such as distention of the stomach and intestinal wall (Fogel et al., 1996), or by chemical stimuli, such as GI peptides (ghrelin, CCK, GLP‐1, and PYY), nutrients and bacterial degradation products (Klingbeil & de La Serre, 2018; Williams et al., 2015). Thus, afferent signals generate satiety by conducting negative feedback between the gut‐brain connection and by regulating digestive reflexes (Schwartz et al., 2000).

GI hormones act in a paracrine way, by locally controlling the functioning of the stomach and intestine and by stimulating the activity of the vagus nerve, and in an endocrine way, by acting directly on its receptors present in the CNS (Moran & Dailey, 2011; Steinert et al., 2017). The intestine is populated by microorganisms, the gut microbiota, which have an impact on the physiology and/or pathophysiology of both animals and humans, particularly regarding inflammation, regulation of energy balance, and obesity (Klingbeil & de La Serre, 2018). The *Firmicutes* phylum is associated with higher obesity risk, whereas *Bacteroidetes* protects against obesity (John & Mullin, 2016). Intestinal bacteria produce several metabolites with physiological activity (Bäckhed et al., 2005; Tseng & Wu, 2019), including SCFAs such as acetate, propionate, and butyrate found in the intestines of humans and rodents. SCFAs can inhibit food intake by binding to GPR41 and GPR43 receptors on intestinal L cells, increasing the secretion of GLP‐1 and PYY (Chambers et al., 2015; Fluitman et al., 2018; Rahat‐Rozenbloom et al., 2017). GPR41 and GPR43 receptors are also expressed in white adipose tissue, immune cells, pancreatic islets, and the CNS (Tang & Offermanns, 2017). In these tissues, SCFAs modulate adipogenesis/lipolysis (Kimura et al., 2014) and the systemic and central immune/inflammatory response (Tang & Offermanns, 2017; Wenzel et al., 2020).

Together, all the aforementioned components constitute the gut‐brain axis; the deregulation of this axis influences the control of food intake and has been seen as one of the factors involved in obesity onset (Bliss & Whiteside, 2018), which is a serious, costly, and multifactorial public health problem that affects the world population regardless of race, sex, or social class (Apovian, 2016; Williams et al., 2015). To investigate obesogenesis, some animal models have been developed, for example, using cafeteria diets, diets rich in sugar or fat, genetic modification, knockout mice, or metabolic programming (Kleinert et al., 2018; Speakman et al., 2008). The metabolic programming model consists of generating a nutritional, environmental, or hormonal insult in critical periods of development, such as pregnancy, breastfeeding, or puberty, causing metabolic changes, which can generate diseases such as obesity in adulthood (Barker, 2004; de Moura et al., 2008). A widely used programming model that causes overweight, abdominal obesity, changes in anorexigenic, and orexigenic neuropeptides, and other metabolic disorders is the litter size reduction after birth (Habbout et al., 2013; Plagemann et al., 1992; Velkoska et al., 2005). Our research group demonstrated that male Wistar rats from reduced litters have hyperphagia, increased body weight, increased total and visceral fat, hypertrophy of adipocytes, increased sympathetic autonomic nervous activity, and lower UCP1 expression, among other outcomes in adulthood (Conceição et al., 2011, 2017; Rodrigues et al., 2009, 2011). We know that these animals have hyperleptinemia and hypothalamic resistance to leptin, which may explain their hyperphagia (Rodrigues et al., 2011).

Therefore, given the phenotype of animals programmed by overfeeding during lactation and the importance of the gut‐brain axis, the hypothesis of this study is that animals raised in small litters develop an imbalance in the gut‐brain axis, leading to hyperphagia. Thus, the aim of this study was to investigate the in vivo electrical activity of the vagus nerve, the expression of c‐Fos in the NTS, the expression of GI hormone receptors in the small intestine or the hypothalamus, the expression of BDNF in the small intestine, the gut microbiota, and the fecal content of SCFAs in male and female rats at 5 months of age programmed by litter size reduction.

## 2 MATERIALS AND METHODS

### 2.1 Ethics and animal procedures

Protocols followed the National Institutes of Health Guide for the Care and Use of Laboratory Animals and the Brazilian Federal Law n° 11.794/2008. Experiments were approved by the Institutional Ethical Committee for the Use of Laboratory Animals of the Biology Institute of the State University of Rio de Janeiro (project authorization number: CEUA/033/2019). Wistar rats were housed in a temperature (22 ± 1°C) and humidity (50%–55%) controlled room on a 12‐h dark‐light cycle, lights on: 7:00 a.m. and lights off: 7:00 p.m., with standard chow (Nuvilab^®^), as depicted in Table 1, and water available ad libitum. Adult virgin female rats were caged with male rats at a ratio of 3:1 for mating once per week. Then, the pregnant rats were individually housed until delivery.

**TABLE 1:** Nutritional composition of the standard chow

Ingredients (g/kg)	
Soybean + wheat	220.0
Corn starch	676.0
Soybean oil	50.0
Vitamin mix^c^	4.0
Mineral mix^c^	40.0
**Macronutrient composition (%)**	
Protein	22.0
Carbohydrate	67.0
Fat	11.0
Total energy (kJ/kg)	17,038.7
**Product enrichment (kg)**	
Vitamin A	25,200 IU
Vitamin D3	2,100 IU
Vitamin E	60 mg
Vitamin K3	12.5 mg
Vitamin B1	14.4 mg
Vitamin B2	11 mg
Vitamin B6	12 mg
Vitamin B12	60 μg
Niacin	60 mg
Pantothenic acid	112 mg
Folic acid	6 mg
Biotin	0.26 mg
Choline	1,100 mg

Note

Standard diet for rodents in pellet format (Nuvilab CR‐1, Nuvital Nutrientes). Minerals (Iron 50 mg; zinc 60 mg; copper 10 mg; iodine 2 mg; manganese 60 mg; selenium 0.05 mg; cobalt 1.5 mg); amino acids (100 mg lysine; 300 mg methionine); additives (antioxidant 100 mg).

### 2.2 Experimental design

**FIGURE 1:**
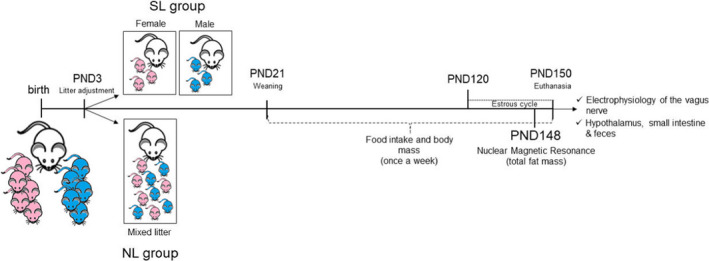
Experimental timeline. Model of litter size reduction. SL, small litter; NL, normal litter; PND, postnatal day; PND3, litter adjustment; PND21, weaning period; PND120 to 150, estrous cycle evaluation; PND148, nuclear magnetic ressonance (NMR) evaluation; PND150, euthanasia of the animals

The study protocol is depicted in Figure 1. To induce early overfeeding, litter size reduction was performed at postnatal day (PND) 3; 30 dams per group were randomly divided into the following groups: male small litter (SL group, *n* = 10) was kept with 3 males; female small litter (SL group, *n* = 10) was kept with 3 females; normal litter (NL group, *n* = 10) was kept with 10 pups (5 males and 5 females) until weaning (3 weeks of age). When necessary, cross‐fostering was done. After weaning, 3–4 animals were kept per cage (same group and sex). Food intake and body mass were monitored weekly until 5 months of age (euthanasia; between 9:00 and 11:00 a.m.). The estrous cycle of SL and NL female offspring was monitored in the last 30 days of life; both groups had regular 4–5‐day cycles. Females were killed during diestrous. The number of rats planned for the analyses was 1 animal/litter/sex. The rest of the animals were used in other studies.

### 2.3 Determination of the total fat mass

Nuclear magnetic resonance (NMR) for small living animals was performed for the evaluation of total fat mass at PND148 using whole‐body composition analyzer NMR equipment (Bruker's Minispec LF90 TD‐NMR), as previously reported (Rodrigues et al., 2018). A quality control check of internal voltages, temperature, magnets, and NMR parameters was performed using a standard provided by the manufacturer. Nonanesthetized rats were placed in a clear, plastic cylinder and kept immobile by insertion of a tight‐fitting plunger into the cylinder. The cylinder was inserted in the equipment chamber for 2 min of scanning. The technician was blind as to group assignment. Data were expressed as % fat mass.

### 2.4 Electrophysiology of the vagus nerve

At PND150, after 12 h of fasting, rats were anesthetized with thiopental (30 mg/kg body mass) for in vivo assessment of autonomic vagus nerve activity. The left vagus nerve was isolated at the level of the left carotid artery in the neck, and electrodes were inserted (Reno et al., 2019). This region is above the superior vagal ganglion, where all afferent and efferent fibers are present (Yuan & Silberstein, 2016). Next, animals were placed inside a Faraday cage to avoid electromagnetic interference; the nerves were placed on a pair of platinum hook electrodes connected to an electronic device (Bio‐Amplifier, Insight^®^) to record the electrical signals. To avoid dehydration, the nerve was covered with mineral oil. Nerve activity was amplified (10,000×) and filtered (cutoff: 60 kHz). A 5‐min period of stabilization and a 10‐min reading period were used. The average number of spikes per 10‐s time window was noted. The background noise level was determined in a nerve segment. More details can be found in the supplementary material. The results were analyzed using the PowerLab data acquisition system (8SP; AD Instruments).

### 2.5 Euthanasia and tissue collection

After reading the nerve activity, the animals were euthanized by cardiac puncture. The visceral fat compartments (retroperitoneal, mesenteric, and gonadal) were collected, weighed, and stored at −80°C. These three depots represent the total visceral fat mass, which was expressed as visceral fat mass/body mass. Subcutaneous adipose tissue was calculated through the difference between the total fat obtained by NMR and the amount of the three visceral compartments.

**FIGURE 2:**
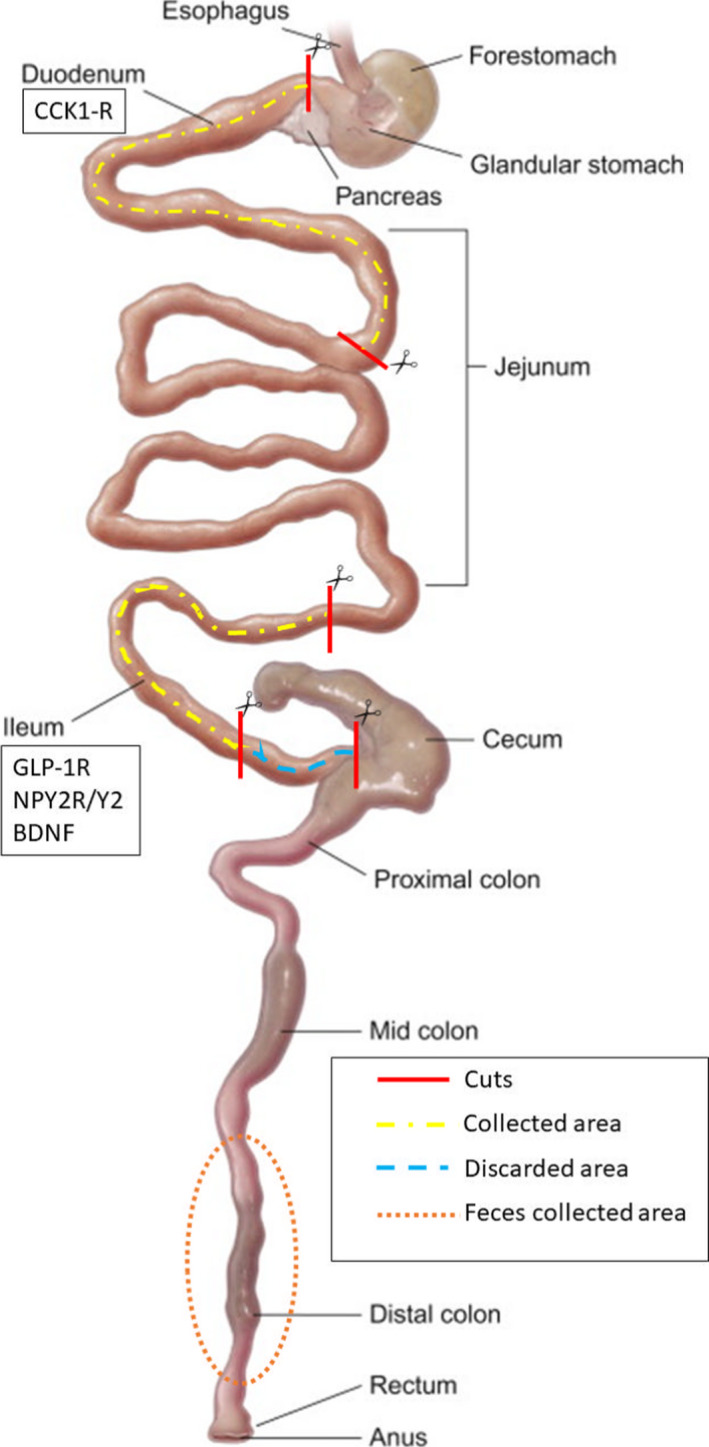
Schematic model of small intestine dissection and stool collection—Scissors with continuous thread, cutpoints; proximal area with dash and dot line (duodenum plus small part of the proximal jejunum) was used to mark CCK1‐R, and the distal area (ileum) was used to mark GLP1‐R, NPY2R/Y2, and BDNF; the tracer line represents the discard area; and dotted circle (distal colon), area in which feces were removed to determine SCFAs and phyla of the microbiota

The small intestine was collected as follows (depicted in Figure 2): The removal of the initial region (duodenum plus small part of the proximal jejunum) was performed from a cut at the height of the pylorus and measured 10 cm in the distal direction for the next cut (to determine CCK1‐R); to collect the ileum most distal region of the small intestine, a cut was made at the ileum cecal junction, and 15 cm was measured, the first 5‐cm closest to the cecum were discarded and the remaining 10 cm were used in the experiment (to determine BDNF, GLP1‐R, and NPY2R/Y2). All regions were washed with 0.9% saline, frozen immediately, and stored in a freezer. Feces used were removed from inside of the distal colon (to determine SCFAs and microbiota phyla). Tissues were stored in a freezer at −20°C.

Isolation of the central regions was performed with coronal sections of the brain generated using a cryostat (Hyrax C52, Zeiss). The coordinates described in the stereotaxic atlas (Paxinos & Watson, 2004) were used for the isolation of the arcuate nucleus (ARC, bregma −2.04 mm to −3.60 mm) and nucleus of the solitary tract (NTS, bregma −12.96 mm to −13.56 mm). The nuclei were then frozen at −80°C for use in western blotting (for additional details, see the supplementary material).

### 2.6 Gas chromatography coupled to mass spectrometry—SCFAs analysis

The protocol was adapted from Pogribna et al. (2008) for measuring SCFAs in the feces. Approximately 350 mg of feces was added to 200 µl of 100 mM formic acid (internal standard; Merck Millipore). Then, 200 µl of 10 mol/L sulfuric acid (Reagen) and 3 ml of ethyl ether (Merck Millipore) were added, mixed for 15 min in an orbital shaker (IKA KS 130 Basic, IKA‐Werke GmbH and Co.) and centrifuged for 5 min at 12,300*g*. The upper phase was reduced under a nitrogen stream, transferred to a flask, and analyzed by gas chromatography coupled to mass spectrometry (GC/MS). GC/MS Shimadzu equipment, model GP2010 Plus, with an Agilent DBWAX (polyethylene glycol) column (60 m × 0.25 mm × 0.25 µm) was used. The injector was kept at 260°C, with a flow division of 20:1. The column temperature was 75°C, maintained for 2 min, and then 75–250°C, with a heating rate of 25°C/min, for 5 min. Helium was used as the carrier gas with a linear velocity of 24.7 cm/s. A volume of 1 µl of the sample was injected into the chromatograph. For the detection by mass spectrometry, a detector containing an electron ionization source (EI‐70 eV) and a quadrupole mass analyzer operated in survey tools from 41 to 301 amu are used. An interface was maintained at 250°C and an ion source at 25°C. The identification of the constituents of the mixture was made by comparing the mass spectra generated with the spectra in the NIST05 library contained in the mass spectrometer computer, as well as with their retention times with the standards of formic acid, acetic acid, propionic acid, and butyric acid.

### 2.7 Real‐time reverse transcription‐polymerase chain reaction (RT‐PCR)—16S rDNA

Approximately 200 mg of feces was used for microbial DNA extraction using a commercial kit (QIAamp Fast DNA stool mini kit, Qiagen) following the manufacturer's instructions. For the reaction, 10 ng of DNA extracted per sample was used plus 6 µl of “SYBR green RT‐PCR mix” (Life Technologies) and 0.2 µmol/L of each initiator: *Eubacteria* (All Bacteria; F:5′‐ACTCCTACGGGAGGCAGCAGT‐3′, R:5′‐ATTACCGCGGCTGCTGGC‐3′); *Bacteroidetes* (F:5′‐CRAACAGGATTAGATACCCT‐3′, R:5′‐GGTAAGGTTCCTCGCGTAT‐3′); *Firmicutes* (F:5′‐TGAAACTYAAAGGAATTGACG‐3′, R:5′‐ACCATGCACCACCTGTC‐3′); class‐γ *Proteobacteria* (F:5′‐ TCGTCAGCTCGTGTYGTGA‐3′, TCCTCCCTG′‐CGTAAGGGCCATGATG‐3′); *Actinobacteria* (F:5′‐TACGGCCGCAAGGCTA‐3′, R:5′‐TCRTCCCCACCTTCCTCCG‐3′). The final reaction volume was 12 µl; water was added to all samples to reach that volume. Samples were used in duplicate and runs used negative controls without the addition of DNA (NTC, from the English “Non‐TemplateControl”) to detect possible contamination of the reaction. RT‐PCR was performed on the StepOnePlus^™^ Real‐Time PCR System (Life Technologies) programmed for the following cycle: 95°C/10 min; 95°C/15 s; and 60°C/1 min (45 cycles). PCR reactions to identify different bacteria phyla were based on previous studies (Rosado et al., 2021; Silva‐Veiga et al., 2020) taking into account the formula provided by Bacchetti De Gregoris et al. (2011) *X* = (Eff. Univ)Ctuniv/(Eff. Spec)Ctspec*100; Where Cts (both universal and specific) are the threshold cycles registered by the thermocycler. Eff. Univ refers to the calculated efficiency of universal primers (2 = 100% and 1 = 0%) and Eff. Spec is the efficiency of the taxon‐specific primers. According to the equation, *X* represents the percentage of 16S taxon‐specific copy numbers in each sample. The evaluation of gut microbiota was presented as a relative abundance.

### 2.8 Western blotting analysis

Protein expression in the small intestine, ARC, and NTS was evaluated by western blotting. For protein extraction, each tissue was homogenized by maceration. After freezing with liquid nitrogen, the macerated tissue was placed in a specific buffer for protein extraction (T‐PER Tissue Protein Extraction Reagent, Thermo Scientific). Inhibitor cocktail (IC) was added at a proportion of 1 μl IC to 100 μl buffer. Small intestine homogenates were centrifuged (12,851, 4°C, 25 min), and then the supernatant phase was collected. The ARC and NTS were sonicated (two pulses of 10 s with 40% amplitude, intercalated by 15 s off). The total protein concentration in homogenates was determined by a Pierce BCA Protein Assay Kit (Thermo Scientific), and analyses of specific proteins (GHS‐R, CCK1‐R, GLP‐1R, NPY2R/Y2, BDNF, and c‐Fos) were performed by SDS‐PAGE. The small intestine proteins were normalized to 20 μg/sample, and the ARC and NTS proteins were normalized to 10 μg/sample. Samples were transferred onto PVDF membranes (Hybond ECL; Amersham Pharmacia Biotech). All membranes were incubated with Tris‐buffered saline (TBS) containing 5% albumin for 45 min. Subsequently, membranes were cut following the molecular weight standard of each protein of interest. Each stretch of the collected membrane was incubated with a specific primary antibody to detect each protein present at different molecular weights; after this, the membranes were washed three times with Tween TBS (0.1%) after the secondary antibodies were used. All antibodies are described in Table 2. After incubation with secondary antibodies, membranes were washed followed by incubation with streptavidin‐conjugated horseradish peroxidase (Caltag Laboratories). Protein bands were visualized by chemiluminescence (Kit ECL plus, Amersham Biosciences) followed by exposure to Image Quant LAS (GE Healthcare). The band area and density were quantified by ImageJ software (Wayne Rasband, National Institutes of Health) and normalized to β‐actin, GAPDH or cyclophilin. Protein expression was quantified using different gels for males and females. Data are expressed as relative (%) to the control group (NL) per sex.

**TABLE 2:** Antibody list

Primary antibodies			Secondary antibodies		
Antibody	Description	Dilution	Specificity	Description	Dilution
Anti‐CCK1‐R	Abcam‐ab77269	1:300	Anti‐goat	Santa Cruz Biotechnology‐sc‐2352	1:5000
Anti‐GLP‐1R	Abcam‐ab218532	1:300	Anti‐rabbit	Sigma‐Aldrich‐SAB4600068	1:5000
Anti‐NPY2R/Y2	Abcam‐ab31894	1:200	Anti‐rabbit	Sigma‐Aldrich‐SAB4600068	1:10,000
Anti‐GHS‐R	Abcam‐ab85104	1:300	Anti‐rabbit	Sigma‐Aldrich‐SAB4600068	1:7000
Anti‐c‐Fos	Santa Cruz Biotechnology‐GW21144	1:500	Anti‐chicken	Sigma‐Aldrich‐A9046	1:7000
Anti‐BDNF	EMD Millipore‐GF35L	1:500	Anti‐mouse	Santa Cruz Biotechnology‐sc‐2377	1:10,000
Anti‐β‐actin	Sigma‐Aldrich‐A2228	1:1000	Anti‐mouse	Santa Cruz Biotechnology‐sc‐2377	1:10,000
Anti‐GAPDH	Cell Signaling Technology‐14C10	1:500	Anti‐rabbit	Sigma‐Aldrich‐SAB4600068	1:10,000
Anti‐Cyclophilin	Cell Signaling Tecnology‐B7389	1:1000	Anti‐rabbit	Sigma‐Aldrich‐SAB4600068	1:10,000

Abbreviations: BDNF, brain‐derived neurotrophic factor; CCK1‐R, Cholecystokinin type‐1 receptor; c‐Fos, proto‐oncogene c‐fos; GHS‐R, Growth hormone secretagogue receptor; GLP‐1R, Glucagon like peptide‐1 receptor; NPY2R/Y2, NPY receptor type 2; β‐actin or GAPDH or cyclophilin were used as controls in western blot.

### 2.9 Statistical analyses

All data are presented as the means ± standard deviation (SD) using GraphPad Prism version 6.0 for Windows (GraphPad Software, Inc.). Only one offspring per litter per group per sex was randomly used for analysis. Males and females were separately analyzed by Student's *t*‐test since our objective was to understand the programming effect in each sex instead of evaluating the interaction. During western blotting, the runs of the gels of males and females were carried out separately. Differences were considered significant when *p* < 0.05.

## 3 RESULTS

### 3.1 Males

#### 3.1.1 Food intake and biometric parameters

As expected, SL males showed higher food intake (+64%, *p* = 0.0006), body mass (+20%, *p* = 0.0002), subcutaneous fat (+54%, *p* < 0.0001), total fat % (+35%, *p* = 0.0002), and visceral fat mass (+47%, *p* = 0.0002) than NL males (Table 3).

**TABLE 3:** Effect of litter size reduction on the biometrical parameters in male offspring at 5‐month‐old

	Male NL rat	Male SL rat
Cumulative food intake (kg)	7.5 ± 1.1	12.3 ± 0.4^#^
Body mass (g)	503.2 ± 17.2	601.9 ± 12.7^#^
Subcutaneous fat (g)	65.9 ± 5.7	101.4 ± 3.8^#^
Total fat (%)	18.6 ± 1.2	25.2 ± 0.6^#^
Visceral fat mass/body mass (g)	5.2 ± 0.3	7.6 ± 0.4^#^

Note

Values are expressed as mean ± SD.

Abbreviations: NL, Normal litter; SL, Small litter.

# *p* < 0.05, statistical significance between SL versus NL (precise *p*‐value is described in the text); *n* = 10 animals from different litters/group.

#### 3.1.2 Electrophysiology of the vagus nerve and c‐Fos content

**FIGURE 3:**
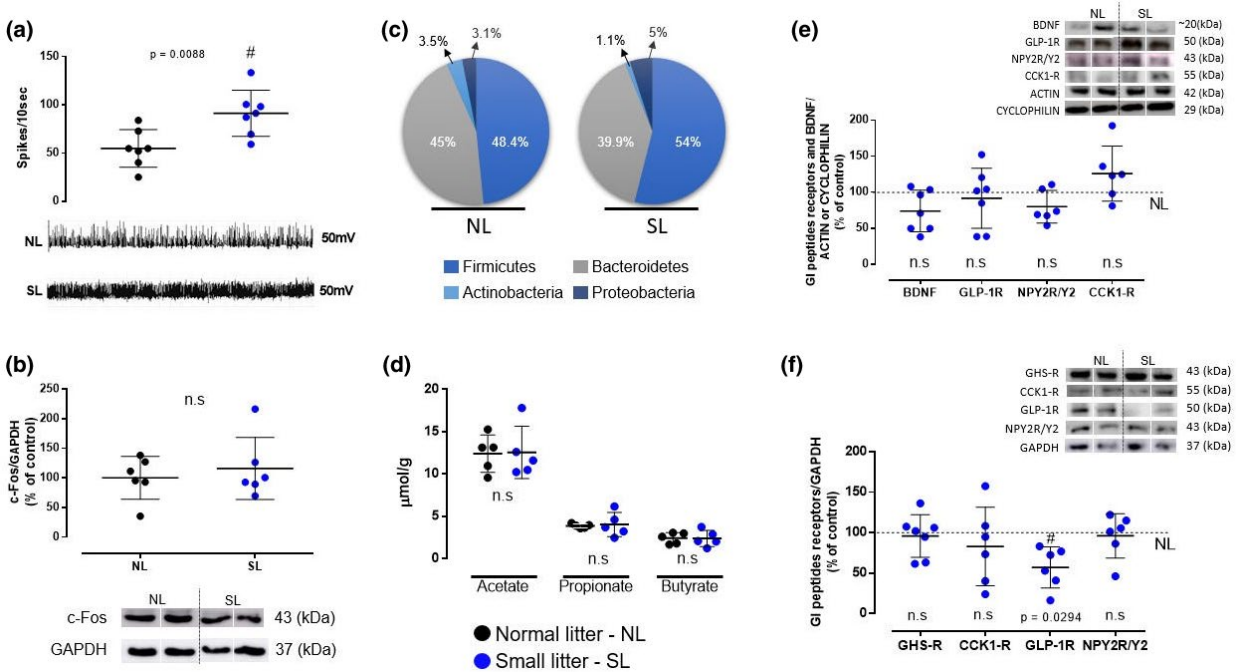
Impact of litter size reduction on markers of the gut‐brain axis in males at 5‐month‐old. (a) Electrical activity of the vagus nerve; images below represent the spikes; *n* = 7 rats per litter/group; (b) Protein expression of c‐Fos in NTS; *n* = 6 rats per litter/group; (c) Relative contribution (%) of four phyla of gut microbiota in the feces; *n* = 4–6 rats per litter/group; (d) Content of SCFAs in the feces; *n* = 5 rats per litter/group; (e) Protein expression of BDNF and GI peptides receptors in the small intestine; *n* = 6–7 rats per litter/group; (f) Protein expression of GI peptides receptors in the hypothalamus; *n* = 5–7 rats per litter/group. NL, normal litter; SL, small litter. Values are expressed as mean ± SD. #*p* < 0.05, statistical significance between SL males versus NL males

SL males had higher vagus nerve activity (+67%, *p* = 0.009) under the basal condition than NL males (Figure 3a). Despite this difference, c‐Fos protein expression in the NTS of SL males was not significantly altered (Figure 3b).

#### 3.1.3 SCFAs and microbiota composition in the feces

The SL group presented a higher abundance of the phyla *Firmicutes* (+19%) and *Proteobacteria* (+62%) but a lower abundance of *Bacteroidetes* (−11%) and *Actinobacteria* (−68%; Figure 3c). This group showed unchanged fecal acetate, propionate, and butyrate contents (Figure 3d).

#### 3.1.4 GI peptides receptors and BDNF

In the small intestine, SL males showed no change in the expression of receptors for intestinal peptides (duodenum) and BDNF (ileum; Figure 3e).

In the hypothalamus, SL males showed lower GLP‐1R protein content (−43%, *p* = 0.03) than NL males (Figure 3f).

### 3.2 Females

#### 3.2.1 Food intake and biometric parameters

For the first time, we are studying the females of this programming model. SL females showed higher food intake (+6%, *p* = 0.005), body mass (+11%, *p* = 0.001), subcutaneous fat (+23%, *p* = 0.02), total fat (+27%, *p* = 0.003), and visceral fat mass (+85%, *p* < 0.0001) than NL females (Table 4).

**TABLE 4:** Effect of litter size reduction on the biometrical parameters in female offspring at 5‐month‐old

	Female NL rat	Female NL rat
Cumulative food intake (kg)	8.3 ± 0.08	8.8 ± 0.12*
Body mass (g)	259.1 ± 3.4	288.6 ± 6.9*
Subcutaneous fat (g)	28.7 ± 1.1	35.4 ± 2.3*
Total fat (%)	13.9 ± 0.4	17.6 ± 0.9*
Visceral fat mass/body mass (g)	2.9 ± 0.1	5.5 ± 0.3*

Note

Values are expressed as mean ± SD.

Abbreviations: NL, Normal litter; SL, Small litter.

* *p* < 0.05, statistical significance between SL versus NL (precise *p*‐value is described in the text); *n* = 10 animals from different litters/group.

#### 3.2.2 Electrophysiology of the vagus nerve and c‐Fos content

**FIGURE 4:**
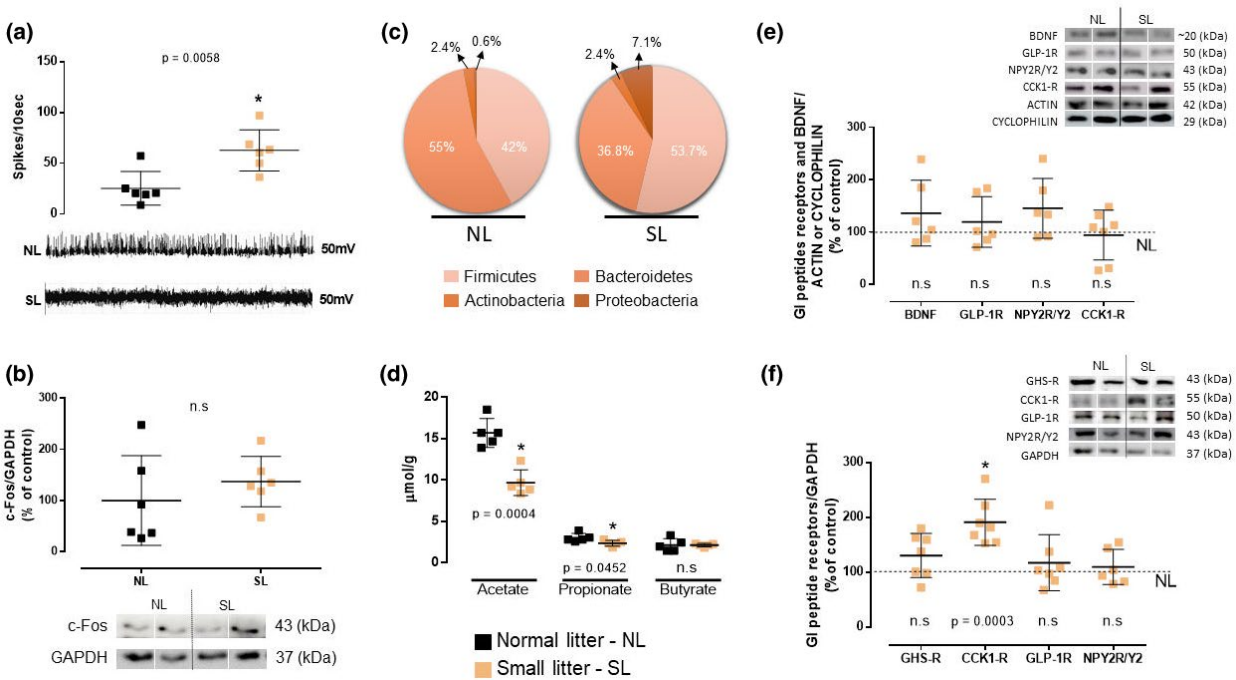
Impact of litter size reduction on markers of the gut‐brain axis in females at 5‐month‐old. (a) Electrical activity of the vagus nerve; images below represent the spikes; *n* = 6 rats per litter/group; (b) Protein expression of c‐Fos in NTS; *n* = 7 rats per litter/group; (c) Relative contribution (%) of four phyla of gut microbiota in the feces; *n* = 6 rats per litter/group; (d) Content of SCFAs in the feces; *n* = 5 rats per litter/group; (e) Protein expression of BDNF and GI peptides receptors in the small intestine; *n* = 6–7 rats per litter/group; (f) Protein expression of GI peptides receptors in the hypothalamus; *n* = 4–7 rats per litter/group. NL, normal litter; SL, small litter. Values are expressed as mean ± SD. *p < 0.05, statistical significance between SL females versus NL females

SL females showed greater basal vagus nerve activity (+148%, *p* = 0.006) than NL females (Figure 4a), while the c‐Fos content in the NTS was not different between the groups (Figure 4b).

#### 3.2.3 SCFAs and microbiota composition in the feces

SL females showed an increase in the abundance of the phylum *Firmicutes* (+ 28%) and an almost 11‐fold increase in *Proteobacteria*, with a reduction in *Bacteroidetes* (−33%) without a difference in *Actinobacteria* (Figure 4c). As depicted in Figure 4d, SL females had lower contents of acetate (−38%, *p* = 0.0004) and propionate (−22%, *p* = 0.045). Fecal butyrate was unaltered between female groups.

#### 3.2.4 GI peptides receptors and BDNF

SL females showed no difference from NL females in the expression of CCK1‐R in the duodenum, GLP‐1R, or NPY2R/Y2 in the ileum or BDNF (Figure 4e).

On the hypothalamus, SL females showed only higher CCK1‐R protein content (+91%, *p* = 0.0003) than NL females (Figure 4f).

## 4 DISCUSSION

The model of litter size reduction during lactation induces increased breast milk consumption by SL offspring, generating an increase in energy intake and, consequently, weight gain (Fiorotto et al., 1991). Moreover, the composition of the milk produced by the SL mother is modified, with an increase in the fat content, mainly triglycerides (Cunha et al., 2009). These two imprinting factors are responsible mainly for causing weight gain and body adiposity accumulation in this programming model. In the present study, neonatal overnutrition due to litter reduction caused an increase in food consumption, body mass, and fat depots in animals, corroborating several data in the literature in males (Habbout et al., 2013; Plagemann et al., 1999; Rodrigues et al., 2009) and the few studies available in females (Costa et al., 2019; Voits et al., 1996). Here, we approach parameters of the gut‐brain axis in this experimental model to understand whether a possible dysfunction of this system can explain hyperphagia. SL animals of both sexes at 5 months of age presented dysbiosis and increased in vivo vagus nerve activity that can contribute to the imbalance of food intake and, consequently, induce weight gain.

In experimental models of obesity induced by diet or postnatal overfeeding, there is an imbalance of the autonomic nervous system, where a sympathetic function is elevated (Conceição et al., 2017; Guarino et al., 2017) and parasympathetic function is reduced (Nagai et al., 2003; Verwaerde et al., 1999). Vagal afferent activity has also been shown to be reduced under a high‐fat diet (Daly et al., 2011). Animals exposed to a high‐fat diet in the perinatal period show reduced excitability of the vagal motoneuron (central efference) of gastric projection and reduced central glutamatergic response (Bhagat et al., 2015), indicating that fat has an important impact on vagal activity. The milk from the SL mother has a higher fat content (Cunha et al., 2009), similar to the milk from mothers fed a high‐fat diet (Bautista et al., 2016). Thus, since the critical period of lactation, when the sensorimotor neural circuits are maturing (Bhagat et al., 2015; Rinaman & Levitt, 1993), it is possible that offspring SL present a failure in the development of the circuitry involved in the vagal reflex. The development of motor‐sensory circuitry depends on BDNF, both at the central level and in peripheral tissues, such as the intestine (Murphy & Fox, 2010). Animals with intestinal BDNF knockout show increased vagal sensory innervation, resulting in reduced mealtime and size (Biddinger & Fox, 2014). In the current study, the intestinal expression of BDNF was not altered in SL animals at 5 months of age, suggesting that the increase in vagal tonus is a mechanism independent of BDNF.

There are still controversies about vagal activity in controlling intake and weight: (1) in humans with morbid obesity, vagus nerve block (vBLOCK therapy) causes weight loss (Camilleri et al., 2008), although this technique, in fact, generates excitability instead of blocking, mimicking the signals sent from the GIT and generating satiety (Pelot et al., 2017); (2) in animals and humans, vagotomy may or may not cause weight loss (Pelot & Grill, 2018); (3) in animals, low‐frequency cervical vagal stimulation results in inhibition of food intake and reduction of body weight (Bugajski et al., 2007; Gil et al., 2009; Val‐Laillet et al., 2010). In our study, the increase in vagal activity may be compensatory to other stimuli for food intake. We suggest that the increase in vagal activity in SL offspring causes a greater release of glutamate. However, as the c‐Fos content in the NTS was not increased and the animals were not hypophagic, possible glutamatergic signaling is reduced in the NTS. Measurement of the glutamate receptor in this tissue can shed light on this mechanism.

The gut microbiota are composed of four major phyla: *Firmicutes*, *Bacteroidetes*, *Proteobacteria*, and *Actinobacteria*; *Firmicutes* and *Bacteroidetes* represent 90% of the bacteria present in mammalian GIT (Shin et al., 2015). In obese humans and animals exposed to a high‐fat diet, there is an increase in the relative abundance of *Firmicutes* compared to *Bacteroidetes* (Horne et al., 2020; Ley et al., 2006). This pattern is also observed in Sprague‐Dawley male rats at 15 days of age raised in small litters (Šefčíková et al., 2011). An increase in the phylum *Proteobacteria* in animals and humans is also related to metabolic disorders (Shin et al., 2015).

This imbalance in the bacterial population characterizes dysbiosis; the increase in intestinal permeability increases the serum concentration of lipopolysaccharide (LPS), generating low‐grade chronic inflammation, which is sufficient to cause metabolic changes that result in increased food intake and weight gain (Cani et al., 2007; Saad et al., 2016). LPS is able to inhibit leptin signaling in vagal afferents (de La Serre et al., 2015). Leptin resistance in the vagus nerve reduces the anorexigenic response of CCK, causing hyperphagia (de Lartigue et al., 2012), and the use of a leptin antagonist in the vagus nerve leads to hyperphagia (Peters et al., 2007). Therefore, according to the pattern of phyla in the fecal microbiota of SL animals of both sexes, we characterized intestinal dysbiosis, possibly leading to low‐grade systemic inflammation and possible inhibition of the leptin effect on the vagus nerve, contributing to the hyperphagia of SL offspring. However, to confirm this hypothesis, specific analyzes of the inflammatory profile are necessary.

There is still disagreement about the relationship between the amount of SCFAs and obesity. Data show an increase in SCFAs in the feces of obese individuals (de la Cuesta‐Zuluaga et al., 2018) and the cecum of obese animals (Turnbaugh et al., 2006). The increase in SFCAs in the cecum was related to dysbiosis and the increase in the *Firmicutes*:*Bacteroidetes* ratio (de la Cuesta‐Zuluaga et al., 2018; Turnbaugh et al., 2006). However, no association between an increase in fecal SFCAs and an imbalance in abundance between *Firmicutes* and *Bacteroidetes* has been shown (Schwiertz et al., 2010). In the present study, the SL males had no alterations of SFCAs in the feces but showed an increase in *Firmicutes* and a reduction in *Bacteroidetes* when compared to controls. Concerning SL females, we found a reduction in acetate and propionate. This finding was similar to the values observed in obese mice fed a high‐fat diet (Murphy et al., 2010). The reduction in acetate and propionate in feces may be related to greater intestinal absorption (1) and/or change in the composition of the microbiota (2), with a reduction in species that produce acetate and propionate. Possibly both situations are occurring. The greater absorption of intestinal SCFAs can impact the positive energy balance of SL females, since SCFAs increase caloric intake in humans and mice (Isken et al., 2010; Riley et al., 2013) and stimulate the accumulation of lipids in adipose tissue (Diamant et al., 2011). The reduction in *Bacteroidetes* may be related to the lower production of propionate due to the reduction in the *Prevotella* genus, which is associated with diets rich in carbohydrates, resistant starches, and fiber and is responsible for the production of large amounts of propionate (Chen et al., 2017; Precup & Vodnar, 2019). In obesity induced by a high‐fat diet or by reduced litter size (male Sprague‐Dawley rats at 15 and 40 days of age), a reduction in the *Prevotella* genus is observed (Marques et al., 2016; Šefčíková et al., 2011). However, obese animals after weight loss showed an increase in *Prevotella*, with a consequent increase in intestinal acetate and propionate (Wang et al., 2020). In addition, the *Firmicutes* phylum is related to greater conversion of acetate to butyrate via the butyryl coenzyme A (CoA): acetate‐CoA transferase pathway (Precup & Vodnar, 2019). Thus, the increase in *Firmicutes* in SL females should reflect acetate reduction. Regarding the effect of SCFAs on GI peptides, in vivo or in vitro administration of SCFAs is known to stimulate the release of PYY and GLP‐1, increasing the serum levels of these anorectic peptides (Tolhurst et al., 2012; Zhou et al., 2008). We suggest that SL females secrete less PYY and GLP‐1 by colonic enteroendocrine L cells, favoring hyperphagia. SCFAs stimulate intestinal transit, while PYY inhibits (Cherbut et al., 1998). In dogs, the reduction in gastric emptying and intestinal motility leads to greater anorexigenic action and reduced food intake (Sallam & Chen, 2011). Thus, if SL females release less PYY, they may have increased intestinal transit, contributing to their hyperphagia.

Locally, GI peptides control food intake (Rodgers et al., 2012) due to their paracrine action on intestinal motility (reducing emptying) and activation of vagal afference (promoting satiety; Krieger, 2020; Lo et al., 2014; Steinert et al., 2017). Obese humans have accelerated gastric emptying, reduced PYY, and increased plasma postprandial GLP‐1 (Acosta et al., 2015). However, male SL animals at 133 days of age had no changes in PYY and GLP‐1 serum levels or the mRNA expression of proglucagon and PYY in the jejunum and colon (Reid et al., 2016). Regarding CCK, Voits et al. (1996) demonstrated that SL animals, regardless of sex, do not present hypophagia after CCK administration. In this sense, the current work measured the expression of GI peptide receptors in the intestine. The protein expression of these receptors was not altered, suggesting that the paracrine action of GI hormones is not programmed by litter size reduction. The fact that we did not measure the serum concentrations of PPY, GLP‐1, CCK and ghrelin may be a limitation of our study.

GI hormones also control appetite by binding to their specific receptors in the hypothalamus (Suzuki et al., 2010). The central role of GLP‐1 has been investigated as a therapeutic target in obesity (Beiroa et al., 2014; Grill, 2020; Terrill et al., 2016). Thus, the reduction of GLP‐1R expression in the ARC of SL males may result in less action of GLP‐1, contributing to the hyperphagic phenotype. The anorexigenic role of CCK involves inhibiting the expression of agouti‐related peptide (AGRP) and neuropeptide Y (NPY; Bi et al., 2004; Dunn et al., 2013). Paradoxically, SL females, who are hyperphagic, show CCK1‐R overexpression in the ARC. Due to dysbiosis, SL females should show increased intestinal permeability, increasing LPS translocation to the blood, which crosses the blood‐brain barrier, causing neuroinflammation (Fuke et al., 2019; Jackson et al., 2019). As LPS upregulates the expression of CCK1‐R in vitro and in vivo (Xu et al., 2004; Zhao et al., 2005), this may be the explanation for the increase in CCK receptors in the ARC of SL females.

It is important to highlight that PCR has inherent limitations, particularly those that result in biases in the template to product ratios of target sequences amplified during PCR from environmental DNA, with such amplification biases found to increase with increasing numbers of PCR cycles. These limitations presented a significant challenge to those who were interested in determining the abundance of individual genes present in the fecal microbiome.

**FIGURE 5:**
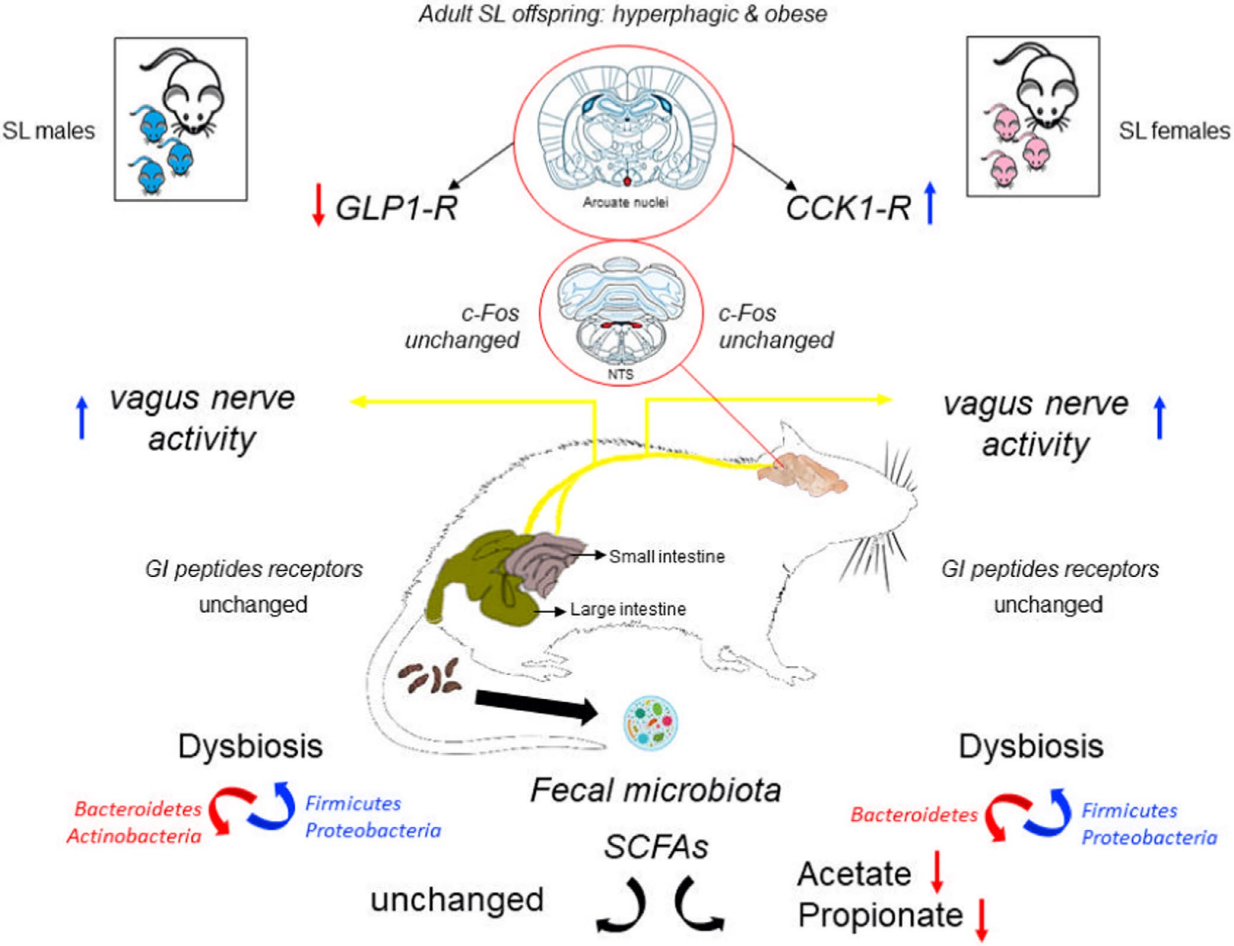
Summary of the main findings. SL, Small litter; Red arrow, decrease; Blue arrow, increase; Arcuate nucleus, red point means collected area; nucleus tractus solitarii (NTS), red points mean collected areas; CCK1‐R, Cholecystokinin type‐1 receptor; GLP‐1R, Glucagon‐like peptide‐1 receptor; GI, Gastrointestinal; c‐Fos, proto‐oncogene c‐Fos; SCFAs, short‐chain fatty acids

In summary, overfeeding during lactation caused by the reduction of litter size caused a disruption in the circuitry of the gut‐brain axis of adult animals of both sexes (Figure 5), contributing to the hyperphagia and obesity already described in the literature. We highlight two important findings in this programming model: (1) The role of signals from the intestine as an important peripheral site for regulating food intake and (2) although few studies have been conducted, SL females present a profile similar to the profile of SL males (hyperphagia, obesity, and gut dysbiosis); however, we evidence a sex dimorphism regarding fecal SCFAs, GLP1, and CCK receptors in the hypothalamus, suggesting dysregulation of the gut‐brain axis. The changes in SCFAs observed only in females could make them more susceptible to negative metabolic outcomes. Thus, feeding dysregulation in early life, mainly due to the consumption of a high‐fat diet, should be avoided to minimize the risk of offspring developing metabolic syndrome in adulthood.

## CONFLICTS OF INTEREST

There is no competing interest that could be perceived as prejudicing the impartiality of the research reported.

## AUTHORS' CONTRIBUTIONS

VST Rodrigues: Conceptualization, Investigation, Methodology, Formal analysis, Writing—original draft; EG Moura: Conceptualization, Visualization, Resources; TC Peixoto: Methodology, Investigation; PN Soares: Methodology, Investigation; BP Lopes: Methodology, Investigation; IM Bertasso: Methodology, Investigation; BS Silva: Methodology, Investigation; SS Cabral: Methodology, Investigation; GEG Kluck: Methodology, Investigation; GC Atella: Methodology, Investigation; JB Daleprane: Methodology, Investigation; PL Trindade: Methodology, Investigation; E Oliveira: Methodology, Investigation, Visualization, Resources; PC Lisboa: Conceptualization, Validation, Formal analysis, Visualization, Resources, Funding acquisition, Data curation, Writing—original draft, Supervision, Project administration.

## ETHICS APPROVAL

Protocols followed the National Institutes of Health Guide for the Care and Use of Laboratory Animals and the Brazilian Federal Law n° 11.794/2008. Experiments were approved by the Institutional Ethical Committee for the Use of Laboratory Animals of the Biology Institute of the State University of Rio de Janeiro (project authorization number: CEUA/033/2019).

## Supporting information



Supplementary Material
