# Supplementary material for: The model of litter size reduction induces long‐term disruption of the gut‐brain axis: An explanation for the hyperphagia of Wistar rats of both sexes
Source: Physiol Rep. 2022 Feb 10;10(3):e15191. doi: 10.14814/phy2.15191 (PMC8831958; doi:10.14814/phy2.15191)
Supplement: Supplementary file 1 — Supplementary Material [file PHY2-10-e15191-s001.docx]

**Supplementary Material - Rodrigues et al**

1. **Vagus nerve activity:**

The Bio-Amplifier device (Insight®, Ribeirão Preto, SP, Brazil) was used to capture the nerve electrical activity and the data were processed in the PowerLab data acquisition system (8SP; AD Instruments, New South Wales, Australia). In this software, the parameters are set to obtain clear and noise-free values.

Steps:

1st - we determine the filtering of the averages;

2nd - we determine that the reading of the peaks beyond the threshold;

3rd - we set the threshold at 0.001v;


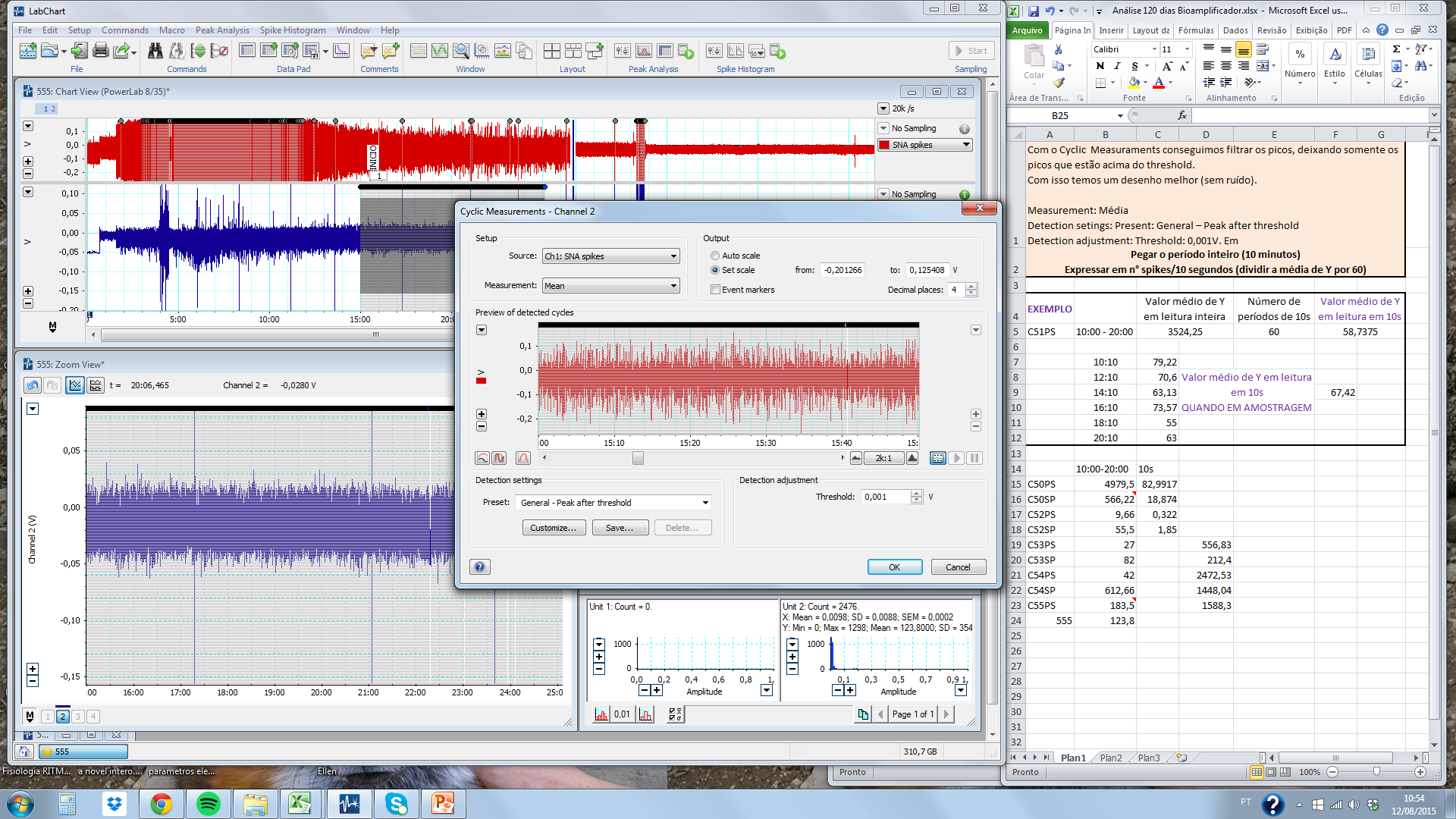


4th - we determine the type of wave extracted.


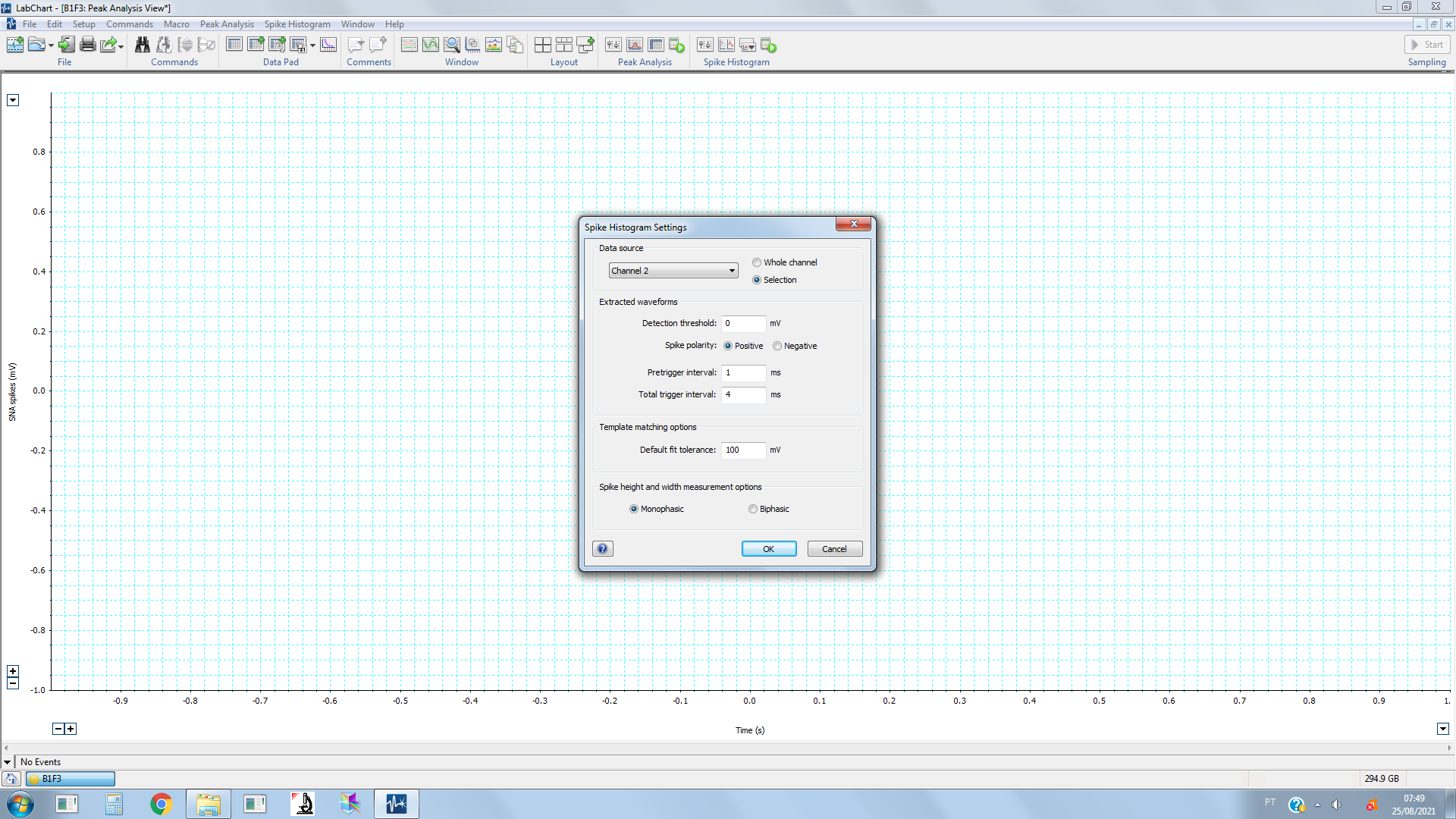


After setting the software, the reading is performed; animals are placed inside a faraday cage to avoid interference during the reading period. The first 5 min are not considered, and from this time onwards, the reading starts (about 20 min), during the reading time, the 10 most homogeneous minutes for quantification are selected.


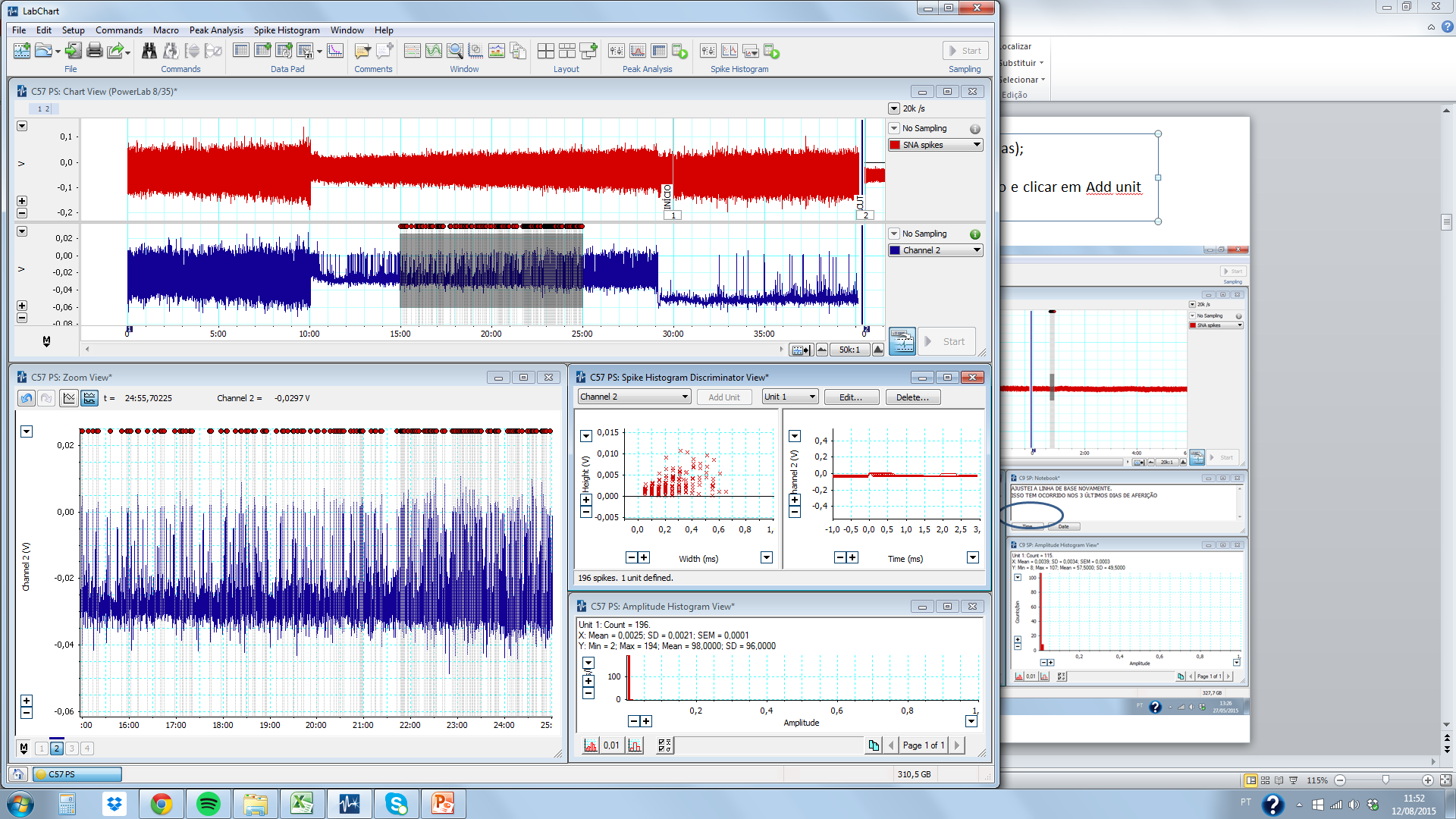


The orange arrow contains filtered peaks (10 homogeneous minutes). In the circle indicated by the blue arrow are the points generated from the spikes; these points will give rise to the amplitude pointed by the pink arrow, which will give the mean value (reading result).

1. **Isolation of the brain nucleus:**

After euthanasia, the brain was quickly dissected and immediately frozen (-20oC) to maintain all anatomical structures. The brains were taken to the cryostat (Hyrax C52, Zeiss, Germany) at -13°C. The location of slices was determined using the stereotaxic atlas (Paxinos and Watson, 2004). Using bregma as a guide, the size of the cuts was determined and, for each cut, a specific needle was used.

ARC: Bregma -2.04 mm to -3.60 mm; the cut size is 1500 µM, the needle used is a square needle positioned in a triangular shape.


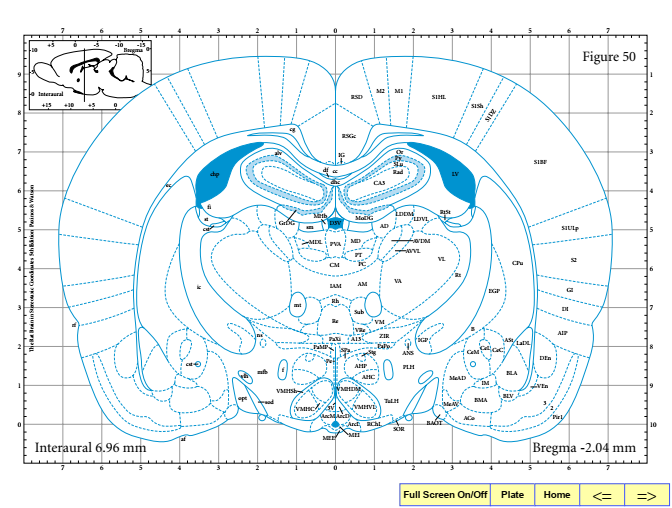


Collected region

NTS: Bregma are -12.96 mm to -13.56 mm, the cut size is 600 µM, the needle used is a 1mm round needle, and the tissue was bilaterally removed.


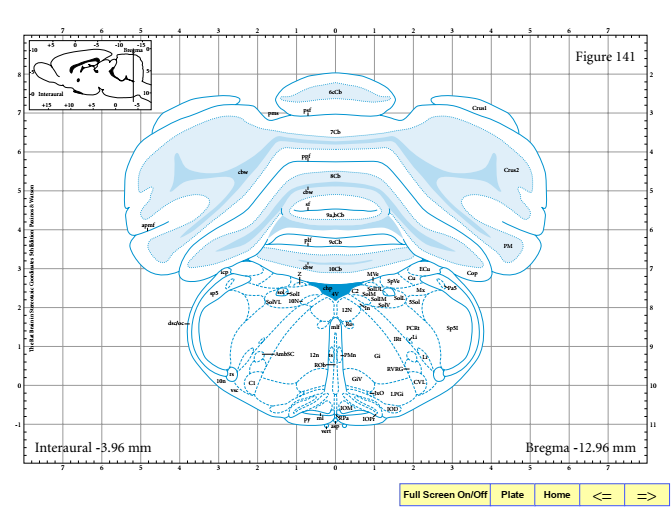


Collected region

1. **Biometric parameters and glycemia:**

Food intake evolution of male rats after weaning period.

Legend: Groups: NL – normal litter, SL – small litter. Values are given as means ± SEM of 10 male cage/group. * (P<0.05); PN=postnatal.

Food intake evolution of female rats after weaning period.

Legend: Groups: NL – normal litter, SL – small litter. Values are given as means ± SEM of 10 male cage/group. * (P<0.05); PN=postnatal.

Body mass evolution of male rats after weaning period.

**Legend:** Groups: NL – normal litter, SL – small litter. Values are given as means ± SEM of 10 male rats/group. * (P<0.05); PN=postnatal.

Body mass evolution of female rats after weaning period.

**Legend:** Groups: NL – normal litter, SL – small litter. Values are given as means ± SEM of 10 female rats/group. * (P<0.05); PN=postnatal.

B)

A)

A)

**Legend:** The effect of postnatal overfeeding on glicemia of males (A) and femles (B) at 5 month-old. Groups: NL – normal litter, SL – small litter. Blue: males; orange: females. Values are given as means ± SEM of 10 rats/group. Unpublished data.
